# Trabecular bone loss in collagen antibody-induced arthritis

**DOI:** 10.1186/s13075-015-0703-5

**Published:** 2015-07-25

**Authors:** Louise Grahnemo, Annica Andersson, Merja Nurkkala-Karlsson, Alexandra Stubelius, Marie K. Lagerquist, Mattias N. D. Svensson, Claes Ohlsson, Hans Carlsten, Ulrika Islander

**Affiliations:** Centre for Bone and Arthritis Research, Department of Rheumatology and Inflammation Research, Institute of Medicine, The Sahlgrenska Academy, University of Gothenburg, Box 480, 405 30 Gothenburg, Sweden; Centre for Bone and Arthritis Research, Department of Internal Medicine and Clinical Nutrition, Institute of Medicine, The Sahlgrenska Academy, University of Gothenburg, Su Sahlgrenska 413 45 Gothenburg, Sweden; Department of Rheumatology and Inflammation Research, Institute of Medicine, The Sahlgrenska Academy, University of Gothenburg, Box 480, 405 30 Gothenburg, Sweden

## Abstract

**Introduction:**

Postmenopausal women with rheumatoid arthritis (RA) have increased risk of developing osteoporosis due to chronic inflammation and estrogen deprivation. Collagen antibody-induced arthritis (CAIA), an experimental polyarthritis model representing the effector phase of arthritis, is mainly mediated by the innate immune system. Compared to the widely used collagen-induced arthritis model, CAIA is conveniently short and can be used in C57BL/6 mice, enabling studies with knock-out mice. However, the impact on bone of the CAIA model in C57BL/6 mice has not previously been studied. Therefore, the aim of this study was to determine if CAIA can be used to study postmenopausal arthritis-induced osteoporosis.

**Methods:**

CAIA was induced by administration of collagen-type II antibodies and lipopolysaccharide to ovariectomized female C57BL/6J mice. Control mice received lipopolysaccharide, but no antibodies. Nine days later, femurs were collected for high-resolution micro-CT and histomorphometry. Serum was used to assess cartilage breakdown and levels of complement. Frequencies of immune cell subsets from bone marrow and lymph nodes were analyzed by flow cytometery.

**Results:**

Trabecular bone mass was decreased and associated with increased number of osteoclasts per bone surface in the CAIA model. Also, the frequency of interleukin-17^+^ cells in lymph nodes was increased in CAIA.

**Conclusion:**

The present study show that CAIA, a short reproducible arthritis model that is compatible with C57BL/6 mice, is associated with increased number of osteoclasts and trabecular bone loss.

## Introduction

Rheumatoid arthritis (RA) is an autoimmune disease in which chronic joint inflammation leads to cartilage and bone destruction. In addition, about 50 % of female postmenopausal RA patients also have generalized osteoporosis [1] and consequently increased risk of fractures. The peak incidence of RA in women occurs at menopause when estrogen levels drop [2, 3] and removal of endogenously produced estrogens by ovariectomy in mice leads to a more severe arthritis and increased bone loss [4]. Collagen-induced arthritis (CIA) is widely used to study arthritis-induced osteoporosis [4–6]. Unfortunately, the susceptibility for CIA is poor in mice of C57BL/6 background, the commonly used strain for knockout models. It is therefore most relevant to find an arthritis model that can be used to study arthritis-induced osteoporosis in C57BL/6 mice. Collagen antibody-induced arthritis (CAIA) is a short commercially available experimental arthritis model representing only the effector phase of arthritis [7] that is mainly mediated by the innate immune system. An intravenous injection of anti-collagen type II (anti-CII) antibodies, directed towards several epitopes on CII in joint cartilage, followed by an intraperitoneal injection of lipopolysaccharide (LPS) rapidly induces polyarthritis. Antibodies bound to cartilage activate the complement system and Fc-receptor-expressing monocytes/macrophages. In addition, neutrophils that produce proteinases and reactive oxygen species are recruited [8–10]. Of note, autoantibodies reactive for CII are also present in a large proportion of RA patients [11]. C57BL/6 mice are susceptible to CAIA, but the development of osteoporosis in C57BL/6 mice with CAIA has never previously been investigated. The aim of this study was thus to determine whether CAIA is a suitable model for studies of postmenopausal arthritis-induced osteoporosis.

## Materials and methods

### Mice

This study was approved by the ethical committee for animal experiments in Gothenburg. Female C57BL/6J mice (Charles River Laboratories, Sulzfeld, Germany) were kept under standard environmental conditions and fed soy-free chow and tap water ad libitum*.* All mice in the experiment, both in the non-arthritic group (“control”, *n* = 14) and in the arthritic group (“CAIA”, *n* = 15), were ovariectomized at 8 weeks of age as described previously [12]. Successful removal of ovaries was confirmed by weighing uteri at termination of the experiment.

### Arthritis induction

Two weeks after ovariectomy, the ArthritoMab™ CII mAb cocktail for C57BL/6 (8 mg/mouse; MD Biosciences GmbH, Zürich, Switzerland) was injected intravenously to induce arthritis (CAIA group). Non-arthritic control mice received phosphate-buffered saline. Three days after antibody administration, 100 μg LPS (*Escherichia coli* 055:B5; MD Biosciences) was injected intraperitoneally to CAIA and control mice. Mice were randomly assigned to experimental groups. The experiment was ended 9 days after antibody administration. This day for termination was chosen based on previous pilot studies showing that arthritis incidence peaked at day 6 after antibody administration and that arthritis severity decreased after day 7.

### Arthritis evaluation

Arthritis incidence and severity were evaluated daily in a blinded manner. Severity was graded 0–3 in each paw (with a total maximum score of 12 per mouse) as follows: swelling in digits: 0.25 points per digit, maximum 1 point per paw; mild, intermediate, or severe swelling in metacarpal/tarsal joints: 0.5, 0.75, or 1 points, respectively; and mild, intermediate, or severe swelling in carpal/tarsal joints: 0.5, 0.75, or 1 points, respectively.

### High-resolution micro-computed tomography

High-resolution micro-computed tomography (μCT) analyses were performed using an 1172 micro-CT model (Bruker, Aartselaar, Belgium) as described previously [12]. Trabecular bone parameters were analyzed in the distal metaphyseal region while the cortical bone parameters were analyzed in the diaphyseal region of femur [12].

### Enzyme-linked immunosorbent assay

Sera were stored at −20 °C until use. Complement factor 3 (C3; Immunology Consultants Laboratory, Inc., Portland, OR, USA), cartilage oligomeric matrix protein (COMP; AnaMar AB, Gothenburg, Sweden), C-terminal telopeptides of type I collagen (CTX-I; Immunodiagnostics Systems Ltd, Boldon, UK), and N-terminal propeptide of type I procollagen (PINP; Immunodiagnostics Systems Ltd) were measured by enzyme-linked immunosorbent assay (ELISA) in serum diluted 1:50,000, 1:10, 1:2, and 1:1, respectively, according to the manufacturer’s instructions. The assay detection limits for C3, CTX-I, and PINP were 1.379 ng/ml, 2 ng/ml, and 7 ng/ml, respectively. The sensitivity of the COMP ELISA was 0.02 U/l.

### Histomorphometry

Tartrate-resistant acid phosphatase activity was demonstrated in femurs as previously described by Toyosawa et al. [13]. The number of osteoclasts (tartrate-resistant acid phosphatase-positive nucleated cells on the bone surface) in the distal femoral epiphysis was counted and divided by the bone surface using a Nikon Eclipse 80i microscope with Osteomeasure™ software (v.3.2.1.7; Osteometrics Inc., Decatur, GA, USA).

### Preparation of cells and flow cytometry analysis

Bone marrow was flushed from the femur using a syringe. Lymph nodes draining the joints were mashed in a 70 μm cell strainer. Single cell suspensions were stained for surface markers using anti-CD3 Horizon V500 and anti-CD11b Horizon V500 (Becton, Dickinson & Company (BD), Franklin Lakes, NJ, USA), anti-CD4 fluorescein isothiocyanate (FITC), anti-MCSF-R allophycocyanin (APC), and anti-F4/80 FITC (BioLegend, San Diego, CA, USA) antibodies. Lymphocytes were gated on singlet cells and CD4^+^ T cells were defined as CD3^+^CD4^+^ cells. Preosteoclasts were gated on singlet cells and defined as CD11b^+^F4/80^+^MCSF-R^+^ cells. For detection of interleukin (IL)-17^+^ cells in lymph nodes, cells were stimulated with phorbol 12-myristate 13-acetate (50 ng/ml; Sigma), ionomycin calcium salt (1 μg/ml; Sigma) and Golgiplug® (BD) for 4 hours at 37 °C and 5 % CO_2_, and stained intracellularly with anti-IL-17A APC (eBioscience, Vienna, Austria). Samples were run on a BD FACS Canto II and data were analyzed using the Flow Jo 8.8.6 or 10.0.6 software (Three Star Inc, Ashland, OR, USA).

### Statistical analysis

Statistical analyses were performed using SPSS software 21.0 (IBM, Armonk, NY, USA). Student’s *t* test was used for comparison of two independent groups. Logarithmic transformations were used when appropriate to ensure normal distribution of data. Experiments were terminated on different days; variation between days was therefore assessed and corrected for when needed using analysis of covariance. The log-rank test was used to analyze arthritis incidence, and data are presented as Kaplan–Meier curves. The area under the curve for arthritis severity versus time was calculated for each mouse by the trapezoidal method:

$$ \mathrm{Area} \approx 0.5\left({y}_0+{y}_1\right)\Delta x + 0.5\left({y}_1+{y}_2\right)\Delta x + 0.5\left({y}_2+{y}_3\right)\Delta x + \dots $$

where ∆*x* is the time between arthritis assessment and *y*_0_, *y*_1_, *y*_2_, *y*_3,_ etc. is the arthritis severity score for day 0, 1, 2, 3, etc. Since the scoring was performed using an ordinal scale, comparisons between groups were analyzed by non-parametric Mann–Whitney test. Differences in *n* are due to lack of sample, sickness, or laboratory errors. All tests are two sided. Data are presented as mean + standard error of the mean, unless otherwise stated. *p* <0.05 was considered significant.

## Results

### Arthritis development in CAIA

**Fig. 1:**
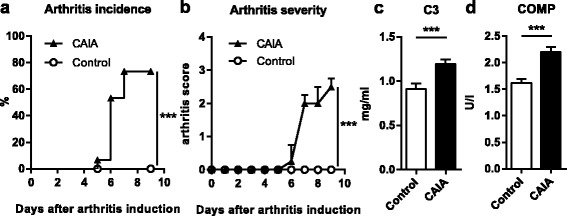
Increased serum levels of C3 and COMP in CAIA mice. C57BL/6 mice were ovariectomized and subjected to LPS + collagen antibodies (CAIA) or LPS alone (control). The experiment was terminated 9 days after arthritis induction. **a** Arthritis incidence in control (*n* = 15) and CAIA (*n* = 15) mice. Log-rank test (***p <0.001). **b** Arthritis severity in control (*n* = 15) and CAIA (*n* = 15) mice. Data are median + upper range. Area under the curve was calculated for each group and analyzed by Mann–Whitney test (***p <0.001). Serum levels of **c** (C3; ***p <0.001) and **d** COMP (***p <0.001) were measured at day 9 after arthritis induction in control (*n* = 14) and CAIA (*n* = 15) mice. Student’s *t* test **c**, **d**, on log data **c**. *C3* complement factor 3, *CAIA* collagen-antibody induced arthritis, *COMP* cartilage oligomeric matrix protein, *LPS* lipopolysaccharide

Arthritis incidence in CAIA mice was 73 %, while no control mice developed arthritis (Fig. 1a). Arthritis severity in CAIA mice was mild and peaked on day 9 (Fig. 1b). Arthritis developed most frequently in the metacarpal/metatarsal and carpal/tarsal joints, and all mice with arthritis had two or more swollen joint regions (metacarpal, metatarsal, carpal, tarsal, and/or one or more swollen digits at day 9) (data not shown). The complement system is important in arthritis development in CAIA [14], and the serum C3 levels were increased by 31 % in CAIA mice compared with controls (Fig. 1c). A common feature of RA is cartilage destruction, and serum levels of COMP—a biomarker of cartilage degradation—were increased by 36 % in CAIA mice compared with controls (Fig. 1d).

### Trabecular, but not cortical, bone loss in CAIA

**Fig. 2:**
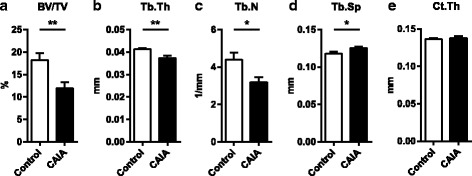
CAIA results in trabecular, but not cortical, bone loss. C57BL/6 mice were ovariectomized and subjected to LPS + collagen antibodies (CAIA) or LPS alone (control). The experiment was terminated 9 days after arthritis induction. **a** Trabecular bone volume as percent of tissue volume (BV/TV) (**p = 0.005), **b** trabecular thickness (Tb.Th) (**p = 0.006), **c** trabecular number (Tb.N) (*p = 0.011), and **d** trabecular separation (Tb.Sp) (*p = 0.024) in the distal metaphyseal region as well as **e** cortical thickness (Ct.Th) (*p* = 0.358) in the diaphyseal region of femur was analyzed by μCT in CAIA (*n* = 7) and control (*n* = 7) mice. Student’s *t* test **a**–**e**, on log data **a**, **b**, **c, e**. *CAIA* collagen-antibody induced arthritis, *LPS* lipopolysaccharide

Generalized bone loss has not previously been studied in the CAIA model. Although the model is very short (9 days), CAIA mice had 34 % lower trabecular bone volume/tissue volume in the distal femoral metaphysis compared with controls (Fig. 2a). Trabecular thickness and trabecular number were decreased by 9 % and 28 %, respectively (Fig. 2b, c), while trabecular spacing was increased by 6 % (Fig. 2d), demonstrating a robust effect of CAIA on trabecular bone loss. However, neither cortical thickness (Fig. 2e) or cortical area in the diaphyseal region of femur (data not shown) was affected in CAIA.

### Increased osteoclast number in femurs of CAIA mice

**Fig. 3:**
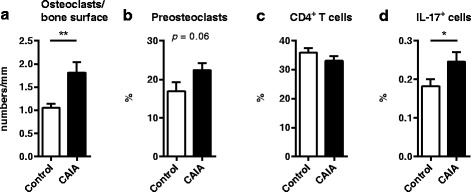
Increase in osteoclasts in CAIA. C57BL/6 mice were ovariectomized and subjected to LPS + collagen antibodies (CAIA) or LPS alone (control). The experiment was terminated 9 days after arthritis induction. **a** Number of osteoclasts (**p = 0.009) in the distal femoral epiphysis per bone surface in control (*n* = 6) and CAIA (*n* = 5) mice, **b** frequency of preosteoclasts (% CD11b^+^F4/80^+^MCSF-R^+^ cells of CD11b^+^ cells) in bone marrow from control (*n* = 11) and CAIA (*n* = 13) mice, **c** frequency of CD4^+^ T cells (of lymphocytes) (*p* = 0.227) in control (*n* = 15) and CAIA (*n* = 14) mice, and **d** frequency of IL-17^+^ cells (of lymphocytes) (*p = 0.049) in control (*n* = 14) and CAIA (*n* = 14) mice, in draining lymph nodes. Student’s *t* test **a**. Analysis of covariance **b**, **c**, and **d,** on log data **b**, **d**. *CAIA* collagen-antibody induced arthritis, *IL* interleukin, *LPS* lipopolysaccharide

A possible explanation for the trabecular bone loss in CAIA is increased number and function of osteoclasts. The number of osteoclasts per bone surface in the distal femoral epiphysis was increased by 81 % in CAIA mice compared with control mice (Fig. 3a). In addition, there was a tendency (*p* = 0.064) for increased frequency of preosteoclasts in the bone marrow of CAIA mice (Fig. 3b).

Although we observed an increase in the number of osteoclasts per bone surface, we did not find any difference in either the bone resorption marker CTX-I (control mice: 28.5 ± 1.4 vs. CAIA mice: 29.4 ± 1.3 ng/ml, *n* = 14–15/group, *p* = 0.643, Student’s *t* test) or the bone formation marker PINP (control mice: 103.3 ± 3.3 vs. CAIA mice: 90.6 ± 7.0 ng/ml, *n* = 14–15/group, *p* = 0.121, Student’s *t* test).

### IL-17^+^ cells are increased in CAIA

T cells, and in particular the Th17 cell associated cytokine IL-17, is involved in the pathology of arthritis-induced osteoporosis [15]. The frequency of T cells and IL-17-producing cells in lymph nodes was therefore analyzed by flow cytometry. The CD4^+^ T-cell frequency was unaffected by CAIA, but IL-17^+^ cells were increased (Fig. 3c, d).

## Discussion

This study reveals for the first time that CAIA can be used as a model to study arthritis-induced generalized bone loss. An increased number of osteoclasts per bone surface in CAIA provides a reasonable explanation for trabecular bone loss. CII antibody deposition in articular cartilage leads to activation of Fc-receptor-expressing cells; for example, macrophages that produce proinflammatory cytokines such as tumor necrosis factor and IL-1β [9]. These cytokines contribute to osteoclast activation and differentiation [16]. Moreover, the Th17-associated cytokine IL-17 induces receptor activator of nuclear factor kappa-B ligand (RANKL) expression on osteoblasts and synovial fibroblasts, resulting in increased *in vitro* osteoclastogenesis [17]. Indeed, in this study increased levels of IL-17^+^ cells were detected in lymph nodes draining the joints in CAIA. The role of IL-17 specifically in CAIA is fairly unstudied; however, IL-17 knockout mice develop less severe arthritis than wildtype controls in the K/BxN serum transfer arthritis model [18]. Induction of CAIA is T-cell independent, although transfer of CII-specific T cells exaggerates arthritis [19]. We thus speculate that Th17 as well as other cells might be producers of IL-17 in CAIA. Indeed, it has become increasingly clear that innate cells can also produce IL-17 in arthritic disease [18, 20]. In addition, the complement system is also important in arthritis development in the CAIA model [14]. Indeed, we found elevated levels of C3 in serum of CAIA mice. Just as IL-17, C3 has been found to be involved in osteoclastogenesis [21], and could thus be another possible mediator of the low trabecular bone in CAIA mice. However, the control mice also had quite high levels of serum C3, possibly explained by the LPS injection, since LPS stimulates C3 synthesis [22]. More studies on cellular and molecular mechanisms of CAIA are thus required to fully understand the link between CII antibody-induced joint inflammation and generalized bone loss.

In contrast to this study, we have previously been unable to detect generalized bone loss in CAIA [23]. However, that study was performed in DBA/1 mice with a different antibody cocktail and bone measurement were performed with peripheral quantitative computer tomography (pQCT). In this study, μCT was used to investigate generalized bone loss, and this technique has also previously been successful to detect local bone erosions and paw swelling in CAIA [24]. CAIA resulted in significant alterations in trabecular bone parameters, but not cortical. The study is probably too short to detect changes in cortical bone, which has a slower bone turnover than trabecular bone [25]. However, the arthritis induced in the CAIA model quickly declines [26] and an extension of the study might therefore not allow establishment of a cortical bone phenotype.

## Conclusion

CAIA is a short convenient model of arthritis-induced osteoporosis in C57BL/6 mice, enabling use of transgenic mice, and serves as a new valuable tool in osteoimmunology research.

## Abbreviations

APC: Allophycocyanin
C3: Complement factor 3
CAIA: Collagen antibody-induced arthritis
CIA: Collagen-induced arthritis
CII: Collagen type II
COMP: Cartilage oligomeric matrix protein
CTX-I: C-terminal telopeptides of type I collagen
ELISA: Enzyme-linked immunosorbent assay
FITC: Fluorescein isothiocyanate
IL: Interleukin
LPS: Lipopolysaccharide
PINP: N-terminal propeptide of type I procollagen
pQCT: Peripheral quantitative computed tomography
RA: Rheumatoid arthritis
RANKL: Receptor activator of nuclear factor kappa-B ligand
μCT: Micro-computed tomography
